# Transient adenovirus-Cre infection causes long-lasting remodeling of the mammary gland immune landscape

**DOI:** 10.1038/s41598-026-43069-8

**Published:** 2026-03-12

**Authors:** Sen Han, Dongyi Zhao, Xueqing Chen, Miao Zhu, Tiantian Li, Chujun Wang, Huabiao Chen, Zhe Li

**Affiliations:** 1 Division of Genetics, Department of Medicine, Brigham and Women’s Hospital, Boston, MA 02115 USA; 2 Department of Medicine, Harvard Medical School, Boston, MA 02115 USA; 3 Vaccine and Immunotherapy Center, Department of Medicine, Massachusetts General Hospital, Charlestown, MA 02129 USA

**Keywords:** Adenovirus-Cre, Mammary gland immune microenvironment, Breast cancer mouse model, p53-loss, CD8+ tissue-resident T cells, Premalignancy, Cancer, Immunology

## Abstract

Understanding how immune cells respond to early oncogenic events is essential for designing immune-based strategies to intercept breast cancer. Mouse models that induce mammary tumorigenesis through Cre-mediated genetic manipulations can be used to study these early events. However, the immune effects of different induction methods remain unclear. Here, we compare adenovirus-delivered Cre with tamoxifen-inducible CreER systems in models targeting luminal mammary epithelial cells for p53-loss. We find that transient intraductal adenoviral infection produces not only an acute immune response but also long-lasting reshaping of the mammary gland immune microenvironment. Adenovirus exposure induces robust and persistent CD8^+^ T-cell infiltration dominated by CD103^+^ tissue-resident T cells displaying heightened activation. This sustained antiviral T-cell signature obscures the p53-loss-driven CD8^+^ T-cell activation detectable in the CreER/tamoxifen model. Adenoviral infection also transiently skews CD4^+^ T cells toward IFN-γ-producing antiviral states and affects the myeloid compartment, whereas tamoxifen-induced p53-loss increases macrophage abundance and activates CD8^+^ T-cells during premalignancy. Despite similar tumor latencies across induction strategies, our findings demonstrate that adenoviral infection exerts long-term immunological effects that can confound interpretation of immune dynamics during early mammary tumorigenesis. These results emphasize the importance of induction-method selection when using genetically engineered mouse models to study cancer-immune interactions.

## Introduction

Development of cancers, such as breast cancer, from their corresponding cellular origins occurs within a dynamic immunological ecosystem in which immune cells can both restrain and promote tumorigenesis. A “fight” between anti-tumorigenic and pro-tumorigenic immune and inflammatory mechanisms can determine the course of tumor development and its phenotype^1,2^. In breast cancer, during its early stages of development, innate and adaptive immune surveillance mechanisms, such as cytotoxic T cells, NK cells, dendritic cells, and macrophages, can recognize and eliminate transformed mammary epithelial cells (MECs), limiting malignant progression^3–5^. However, as oncogenic signaling, genomic instability, and tissue remodeling intensify, the evolving tumor microenvironment can reshape these immune populations, such as during the transition from the precancer to cancer stages^6^. Myeloid-derived immune cells, particularly tumor-associated macrophages and neutrophils, may adopt pro-tumorigenic phenotypes that support immunosuppression, angiogenesis, and invasion, while regulatory T cells and exhausted T-cell states dampen effective antitumor immunity^7–9^. Defining how these immune populations are recruited, activated or suppressed, and reprogrammed during the early phases of mammary tumorigenesis is essential for designing strategies that bolster endogenous immune defenses. By understanding the immunological events that tip the balance from elimination to escape, immune-based preventive approaches may be developed to intercept breast cancer at its roots, before clinically detectable disease emerges.

As access to early stages of human cancer development, including that of breast cancer, is very difficult if not impossible, in order to achieve the goal of immune-based cancer interception, animal models that can recapitulate this early phase of tumorigenesis are essential. In breast cancer, several elegant approaches have been developed to model mammary tumor initiation from a defined subpopulation of MECs and to follow their progression in mice^10^. One of the prevalent approaches is the Cre/loxP-based strategy that enables precise temporal and spatial control over oncogenic events within the mammary epithelium^11^. By placing Cre recombinase under the control of mammary-specific promoters, such as *MMTV*, *Wap*, or *Krt8*^12,13^, and activating it through ligand-dependent systems such as CreER (Cre-estrogen receptor fusion, inducible by tamoxifen^14,15^), researchers can trigger defined genetic alterations, including oncogene activation or tumor suppressor loss, at selected MEC subpopulations or developmental stages. This approach faithfully recapitulates the stepwise evolution of breast cancer, permitting the study of early cellular transformation, clonal expansion, and cancer-immune cell interaction within an intact immune microenvironment^16–19^. To complement the CreER/tamoxifen-based inducible approach, my group developed a novel approach based on intraductal injection of Cre-expressing adenovirus to mouse mammary glands^20–22^. Adenovirus is a DNA virus that does not integrate into the host genome and the adenoviral vector we use only leads to transient expression of the Cre recombinase; thus, it serves a similar purpose as an inducible Cre system. The cell type-specificity (for inducing Cre-mediated recombination) is achieved by using different MEC subpopulation-specific promoters (e.g., *Krt8*, *Wap*) to drive adenovirus-Cre expression.

Breast cancer mouse models based on both CreER/tamoxifen and adenovirus-Cre-based approaches offer excellent in vivo systems to characterize immune cell phenotypes and their dynamic changes at different stages of mammary tumorigenesis. In these induced mice, mammary gland immune cells can be modulated by signals from mutated MECs. However, it is unclear whether the injected tamoxifen or adeno-Cre virus (to induce Cre-mediated recombination and initiation of mammary tumorigenesis) can affect the immune microenvironment. This is possible as administration of tamoxifen in vivo can potentially affect estrogen signaling in the mammary gland^23,24^, while estrogen/ER (estrogen receptor) signaling plays a key role in regulating mammary gland immune cell populations^25^. Similarly, injection of adenovirus to mammary glands should trigger virus-induced immune reactions, at least transiently^20^. However, it is unclear whether any of these induction approach-related modulations of mammary gland immune cells is transient or more long-lasting and whether such change would affect immune cell phenotypes and dynamics, and ultimately, mammary tumor development. A thorough understanding of these should have important implications for the proper use of these modeling approaches to study immune cells during mammary tumorigenesis.

## Results

### Adenoviral infection increases leukocyte infiltration in the mammary gland

In this study, we used a breast cancer mouse model that we previously developed based on induced loss of p53 in luminal MECs^17^. The experimental mice (*PY*) carry *Trp53* conditional knockout alleles (*Trp53*^*fl/fl*^)^26^ along with a conditional Cre-reporter [*Rosa26-LSL-YFP* (*R26Y*)]^27^, while *R26Y* reporter-only mice served as wild-type (WT) controls (Supplementary Fig. 1a). To induce mammary tumor initiation specifically in luminal MECs, we utilized an adenovirus expressing Cre under the control of the luminal *keratin 8* (*Krt8*, or *K8*) promoter (*Ad-K8-Cre*)^20^. Intraductal injection of *Ad-K8-Cre* into the abdominal (#4) mammary glands of *PY* female mice induced simultaneous YFP reporter expression and *Trp53* deletion in luminal MECs, resulting in the development of mammary tumors (predominantly of the Claudin-low subtype) with 100% penetrance^17^. For comparison, we also used a CreER/tamoxifen-based approach to induce p53-loss in the same luminal MEC population. We generated *KPY* experimental mice and *KY* controls by crossing the *K8-CreER* allele, a transgenic construct expressing CreER^T2^ under the same *K8* promoter^12^, with *PY* and *R26Y* mice, respectively (Supplementary Fig. 1b). Tamoxifen (TAM) induction in *KPY* female mice led to development of mammary tumors similar to those observed in *Ad-K8-Cre*-induced *PY* mice, also with full penetrance^17^.

**Fig. 1:**
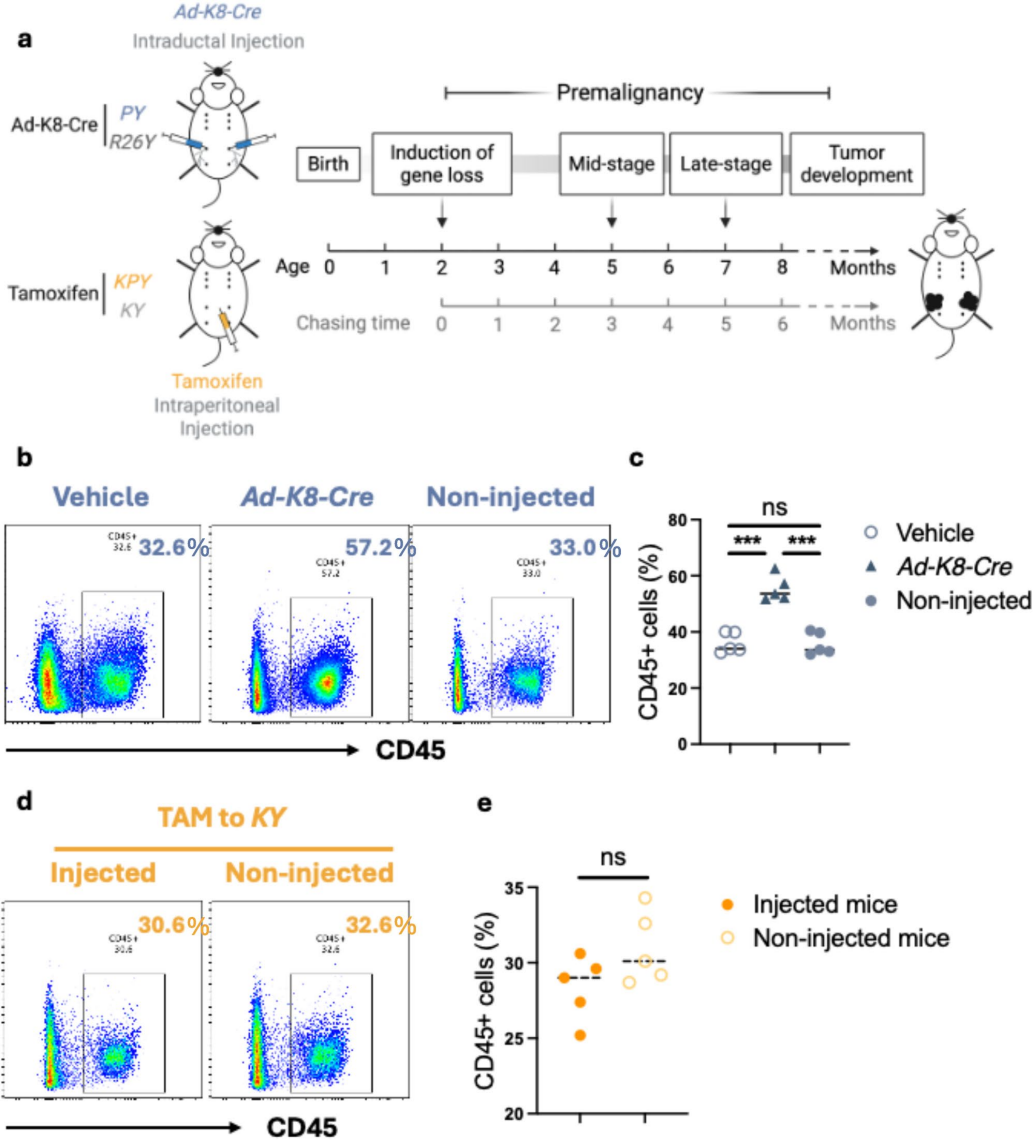
Adenoviral infection enhanced mammary gland leucocyte infiltration. (**a**) Schematic diagram of lineage-tracing schemes using Cre-expressing adenovirus (*Ad-K8-Cre*) or tamoxifen (TAM). (**b**) One week post intraductal injection with *Ad-K8-Cre*, FACS analysis of infiltrated leukocytes (CD45^+^) in the mammary glands with and without (i.e., vehicle or non-injected control) adenovirus injection in *R26Y* mice (n = 5, representative FACS plots are shown). (**c**) Statistical analysis of leukocyte infiltration among the *Ad-K8-Cre*-injected (abdominal) and non-injected mammary glands (thoracic) from the same *R26Y* mice, and vehicle-injected abdominal mammary glands from separate *R26Y* mice. (**d**, **e**) FACS and statistical analysis of leucocyte infiltration in mammary glands of *KY* mice with or without TAM injection (at one week after injection, n = 5). *P* value: ***p < 0.005, ns = not significant. Data represent mean ± S.E.M.

To investigate how p53-loss in luminal MECs alters the mammary gland immune microenvironment during the premalignant stage, we induced p53 deletion by administering *Ad-K8-Cre* or TAM to *PY* (and *R26Y*) or *KPY* (and *KY*) female mice at ~ 8 weeks of age, respectively. Based on our previous findings, mammary tumors begin to emerge in induced mice around 5–6 months after induction^17^. Accordingly, in this study we defined 3 and 5 months post-induction as the mid- and late-premalignant stages, respectively (Fig. 1a). To determine whether intraductally delivered adenovirus itself elicits additional immunogenicity capable of altering the mammary immune microenvironment, we evaluated leukocyte infiltration one week after injection of either adenovirus or vehicle control (i.e., virus dilution buffer only). FACS analysis revealed that adenovirus-injected *R26Y* mice exhibited a significantly higher proportion of CD45^+^ leucocytes in the injected abdominal mammary glands (55.5% ± 7.1%) compared with non-injected thoracic glands from the same mice (35.76% ± 4.74%). Importantly, no significant difference in leukocyte infiltration was observed between vehicle-injected and non-injected glands (Fig. 1b-c), indicating that the altered immune microenvironment in adenovirus-injected glands was attributable to viral infection rather than to the injection procedure itself. In contrast, the *K8-CreER*-based model relies on systemic TAM administration via intraperitoneal injection, which could in principle exert global effects on all mammary glands through its modulation of estrogen signaling. We compared leukocyte infiltration in mammary glands from TAM-treated *KY* mice with that of age-matched, untreated *KY* controls. The data indicate that TAM treatment does not significantly alter leukocyte infiltration (28.36% ± 3.16% vs. 30.98% ± 3.32%) (Fig. 1d-e). As p53-loss is induced in only a small number of luminal MECs in both the adenovirus-induced and TAM-induced models, p53 deletion at this early stage is unlikely to significantly alter the immune microenvironment relative to the effects of adenovirus or tamoxifen administration. Therefore, we did not assess the impact of induced p53 loss on mammary gland immune cells at this initial time point.

### Transient adenoviral infection in the mammary gland increases T cell infiltration

**Fig. 2:**
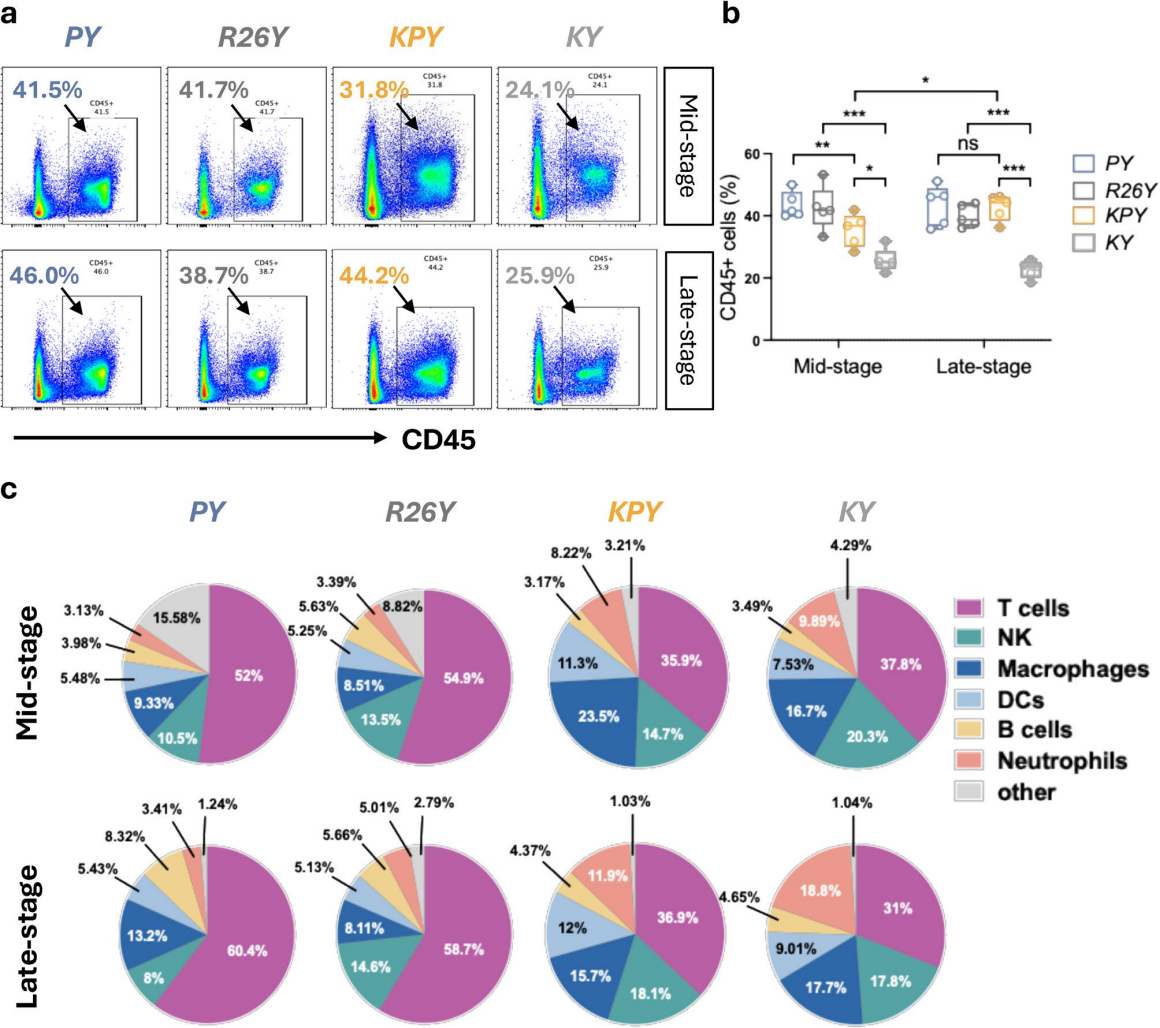
Transient adenoviral infection led to T cell infiltration throughout the premalignant stage. (**a**) Representative FACS plots showing CD45^+^ leucocyte infiltration within the abdominal mammary glands at mid- (upper panel) and late-premalignant (lower panel) stages; dead cells were excluded from the parental gate. (**b**) Statistical analysis for leucocyte infiltration as shown in a (n = 5). (c) Representative pie chart for the composition of major immune cell populations, including T cells (CD45^+^CD3^+^), NK cells (CD45^+^CD3^-^NKp46^+^), Macrophages (CD45^+^CD11b^+^F4/80^+^), dendritic cells (CD45^+^CD11b^+^CD11c^+^), B cells (CD45^+^CD19^+^), and neutrophils (CD45^+^CD11b^+^Ly-6G^+^), within the leucocyte compartment of mammary glands. *P* value: *p < 0.05, **p < 0.01, ns = not significant. Data represent mean ± S.E.M.

To determine whether transient adenoviral infection in *PY* and *R26Y* mice has any long-term effects on the immune microenvironment of the injected mammary glands, we examined leucocyte infiltration at mid- and late-premalignant stages using FACS analysis. At the mid-stage, both adenovirus-infected *PY* and *R26Y* mice displayed higher levels of leukocyte infiltration compared with their counterparts induced via TAM (i.e., *KPY* and *KY*) (Fig. 2a-b). Induced p53 loss in luminal MECs modestly increased immune activity in the mammary glands of *KPY* mice relative to *KY* controls; however, no such difference was detected between adenovirus-induced *PY* and *R26Y* mice (Fig. 2a-b). By the late stage, leukocyte infiltration in induced *KPY* mice rose to levels comparable to those of adenovirus-induced *PY* and *R26Y* mice, whereas *KY* controls remained substantially lower. In contrast, leukocyte infiltration in adenovirus-induced *PY* and *R26Y* mice remained similar to each other throughout both stages (Fig. 2a-b). These findings indicate that transient adenoviral infection causes a sustained increase in mammary gland leukocyte infiltration. Furthermore, loss of p53 in luminal MECs likely triggers an anti-tumor immune response that enhances mammary gland immune activity. This effect is readily detectable in TAM-induced *KPY* mice but may be obscured in adenovirus-induced *PY* mice due to the elevated baseline immune infiltration caused by prior adenoviral exposure.

To exclude the possibility that the increased immune activation observed in the *KPY* model following TAM induction was simply due to a higher recombination efficiency compared with adenoviral delivery, we evaluated the initial induction and subsequent expansion of premalignant MECs in both models. We first quantified the proportion of premalignant cells (YFP^+^) within the lineage-negative compartment (Lin^-^, i.e., CD45^-^TER119^-^CD31^-^) two weeks after induction. This time point was selected to enable comparison of recombination efficiency, as previous studies have shown that Cre recombinase activity can persist for couple weeks following induction in both adenovirus-Cre and CreER/TAM-based systems^20,28^. The proportion of YFP^+^ premalignant cells was comparable between experimental and control groups across both induction strategies (Supplementary Fig. 2a-b). Moreover, more than 90% of YFP^+^ cells localized to the luminal MEC gate (Lin^-^CD24^high^CD29^low^), indicating that both induction approaches achieved similar levels of p53 deletion specifically within luminal MECs (Supplementary Fig. 2c). We next assessed the expansion capacity of p53-null MECs during premalignancy by FACS. In the adenovirus-induced model, p53-null MECs (YFP^+^) represented 0.29% ± 0.13% of the Lin^-^ compartment at two weeks post-induction, increasing to 0.85% ± 0.3% at the mid-stage and 2.89% ± 0.92% at the late-stage of premalignancy. In contrast, YFP^+^ MECs in induced *R26Y* mice (which retain WT p53) remained at a steady percentage (~ 0.6% YFP^+^) throughout the premalignant period (Supplementary Fig. 2 d). A similar pattern was observed in the TAM-induced model (Supplementary Fig. 2e). Notably, the kinetics of premalignant MEC expansion appeared modestly different between the two models (Supplementary Fig. 2d-e). However, as both models rely on a single YFP reporter to mark p53-deficient MECs, differences in clonal dynamics cannot be resolved and would require alternative lineage-tracing approaches. Nevertheless, these results demonstrate that both induction strategies generate comparable initial recombination efficiencies in luminal MECs, and that p53-loss confers a consistent clonal expansion advantage during premalignancy.

To evaluate how transient adenoviral infection reshapes the immune landscape of the mammary gland, we quantified major immune cell populations, including T cells (CD45^+^CD3^+^), NK cells (CD45^+^CD3^-^NKp46^+^), macrophages (CD45^+^CD11b^+^F4/80^+^), dendritic cells (CD45^+^CD11b^+^CD11c^+^), B cells (CD45^+^CD19^+^), and neutrophils (CD45^+^CD11b^+^Ly-6G^+^), by FACS analysis (Supplementary Fig. 3). Across all time points, adenovirus-infected mammary glands in both *PY* and *R26Y* mice exhibited a marked increase in T cells (Fig. 2c), indicating that the elevated baseline immune infiltration observed following adenoviral injection is driven primarily by T cell enrichment.

### Adenoviral infection induces CD8^+^ tissue-resident T cells in the mammary gland

**Fig. 3:**
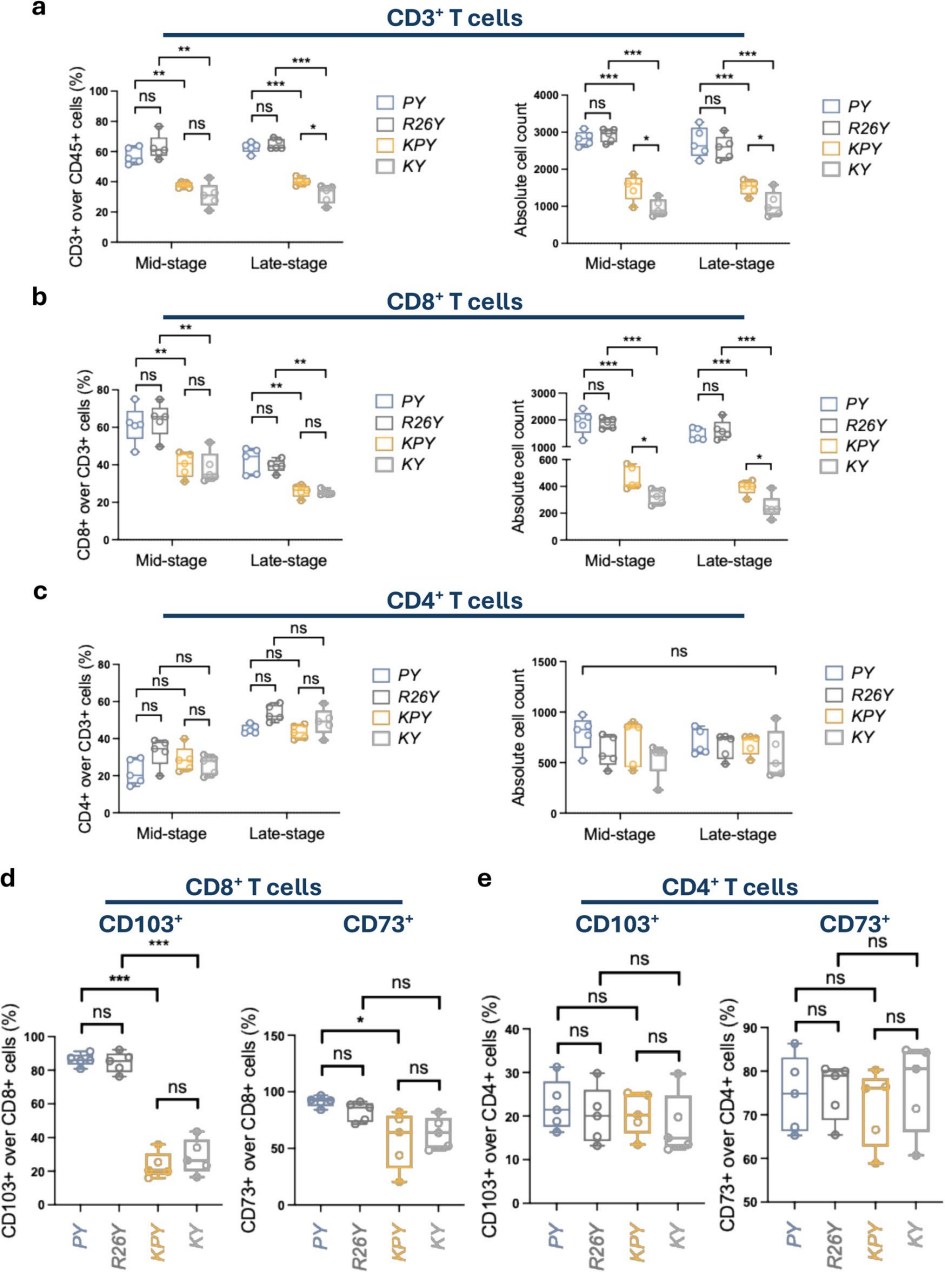
Adenoviral infection resulted in abundant CD8^+^ tissue-resident T cells. Statistical plots for FACS analysis of mammary glands from female mice with the indicated genotypes 3 months and 5 months after inductions. (**a**) Quantification of percentages (left panel) and absolute cell number (right panel) of CD3^+^ T cells within the CD45^+^ compartment of mammary glands from mice with the indicated genotypes (n = 5 each). (**b** and **c**) Quantification of percentages (left panel) and absolute cell number (right panel) of CD8^+^ (b) and CD4^+^ (c) T cells within the CD45^+^CD3^+^ compartment (n = 5). (**d** and **e**) Statistical plots for CD103 and CD73 expression in CD8^+^ and CD4^+^ T cells at 3 months after induction (n = 5). *P* value: *p < 0.05, **p < 0.01, ***p < 0.005, ns = not significant. Data represent mean ± S.E.M.

We next examined the CD3^+^ T cell compartment in greater detail. Regardless of the p53 mutagenesis status, adenoviral infection markedly increased CD3^+^ T cell infiltration, both in proportion level and absolute cell number, in virally infected mammary glands at both mid- and late-premalignant stages, compared with TAM-induced mice (Fig. 3a). This elevation in T cell abundance was driven primarily by an increase in CD8^+^ T cells, rather than CD4^+^ T cells (Fig. 3b-c). Given that transient adenoviral infection produced a sustained elevation in CD8^+^ T cells, we investigated whether this reflected an expansion of tissue-resident memory T cells. To do so, we assessed CD103 and CD73 expression, canonical markers of tissue-resident memory T cells^29,30^, on both CD8^+^ and CD4^+^ T cell populations. In mice analyzed 3 months post-induction, ~ 90% of infiltrating CD8^+^ T cells in adenovirus-infected mammary glands (both *PY* and *R26Y*) expressed CD103, compared with only ~ 30% in TAM-induced *KPY* and *KY* mice (Fig. 3d). CD73 expression was relatively high on CD8⁺ T cells across all groups (Fig. 3d). In contrast, neither CD103 nor CD73 showed substantial differences on CD4^+^ T cells across induction methods (Fig. 3e). Collectively, these findings indicate that transient adenoviral infection elicits a robust CD8^+^ T cell response, promoting their infiltration and long-term residency within the mammary gland microenvironment.

### CD8^+^ tissue-resident T cell phenotype masks p53 loss-driven CD8^+^ T cell activation

**Fig. 4:**
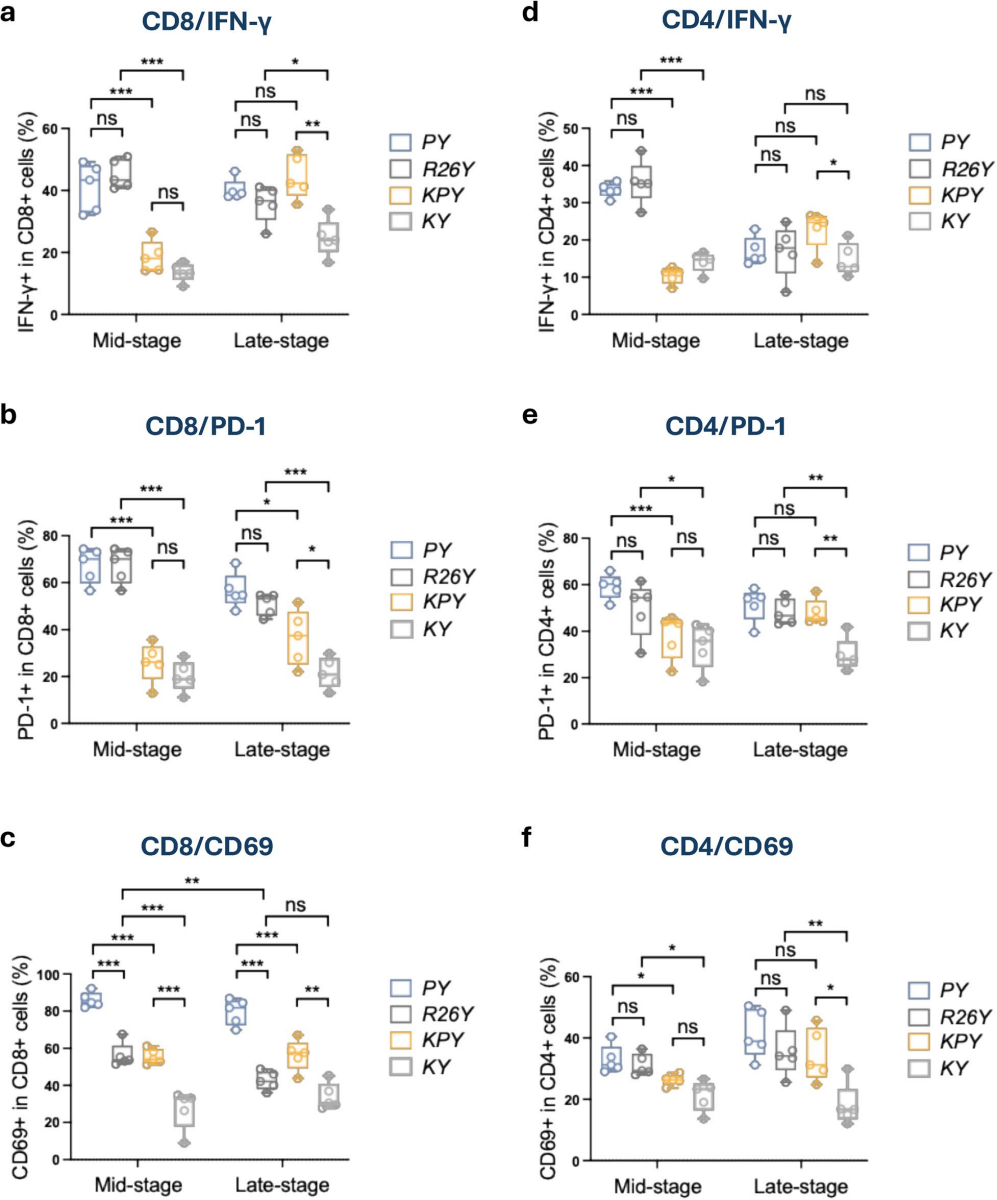
The adenoviral infection-driven CD8^+^ tissue-resident T cell phenotype masked p53 loss-driven CD8^+^ T cell responses. Statistical plots for FACS analysis of mammary glands from female mice with the indicated genotypes 3 and 5 months after inductions. (**a**-**c**) Statistical plots for IFN-γ, PD-1, and CD69 expression in CD8^+^ T cells (n = 5). (**d**-**f**) Statistical plots for IFN-γ, PD-1, and CD69 expression in CD4^+^ T cells (n = 5). *P* value: *p < 0.05, **p < 0.01, ***p < 0.005, ns = not significant. Data represent mean ± S.E.M.

We next characterized CD8^+^ and CD4^+^ T cell subsets and functional states during premalignancy by assessing expression of IFN-γ, as well as the activation markers PD-1 and CD69, using FACS analysis. In the TAM-induced model (*KPY*), induced p53-loss in luminal MECs elicited a pronounced increase in IFN-γ, PD-1, and CD69 expression in CD8^+^ T cells at the late-premalignant stage. At the mid-stage, CD8^+^ T cells in *KPY* mice showed increased CD69 expression but no significant elevation in IFN-γ or PD-1 (Fig. 4a-c). In contrast, in the adenovirus-induced model, induced p53-loss in luminal MECs from *PY* mice did not significantly alter IFN-γ or PD-1 expression in CD8^+^ T cells when comparing to induced *R26Y* controls (Fig. 4a-c). Instead, CD8^+^ T cells in both adenovirus-induced *PY* and *R26Y* mice displayed consistently elevated activation states at both mid- and late-stages relative to their TAM-induced counterparts (Fig. 4a-c). This sustained activation profile appears to result from the large pool of tissue-resident CD8^+^ T cells resulted from the prior adenoviral infection. Supporting this, the majority of PD-1^+^CD8^+^ and CD69^+^CD8^+^ cells in adenovirus-induced *PY* and *R26Y* mice co-expressed CD103, whereas most PD-1^+^CD8^+^ and CD69^+^CD8^+^ cells in TAM-induced *KPY* and *KY* mice lacked CD103 expression (Supplementary Fig. 4a). In contrast, the CD103 expression in the PD-1^+^CD4^+^ and CD69^+^CD4^+^ cells from both models was low and not significantly different (Supplementary Fig. 4b).

In the CD4^+^ T cell compartment, adenovirus-induced *PY* and *R26Y* mice exhibited a transient increase in IFN-γ-producing, activated CD4^+^ T cells at the mid-premalignant stage, which declined to levels comparable to those of TAM-induced mice by the late stage (Fig. 4d). In contrast, mammary glands from TAM-induced *KPY* mice displayed a selective increase in IFN-γ-producing, activated CD4^+^ T cells during the late-premalignant stage compared with *KY* controls, a feature not observed in the adenovirus-induced model (Fig. 4d). A similar late-stage increase in PD1^+^CD4^+^ and CD69^+^CD4^+^ T cells was also uniquely observed in induced *KPY* mice (Fig. 4e-f). Taken together, these findings indicate that transient adenoviral infection recruits CD8^+^ T cells into the injected mammary gland, where they differentiate into tissue-resident T cells. These virus-induced CD8^+^ T cells display a persistently activated phenotype that likely obscures the CD8^+^ T cell response elicited by the expanding p53-null mutant MEC population. In parallel, viral infection appears to drive otherwise quiescent CD4^+^ T cells into a transient, IFN-γ-producing, virus-specific state, which may limit their engagement in immune responses against p53-null MECs.

### Adenoviral infection-induced CD8^+^ T cell influx affects the myeloid compartment

**Fig. 5:**
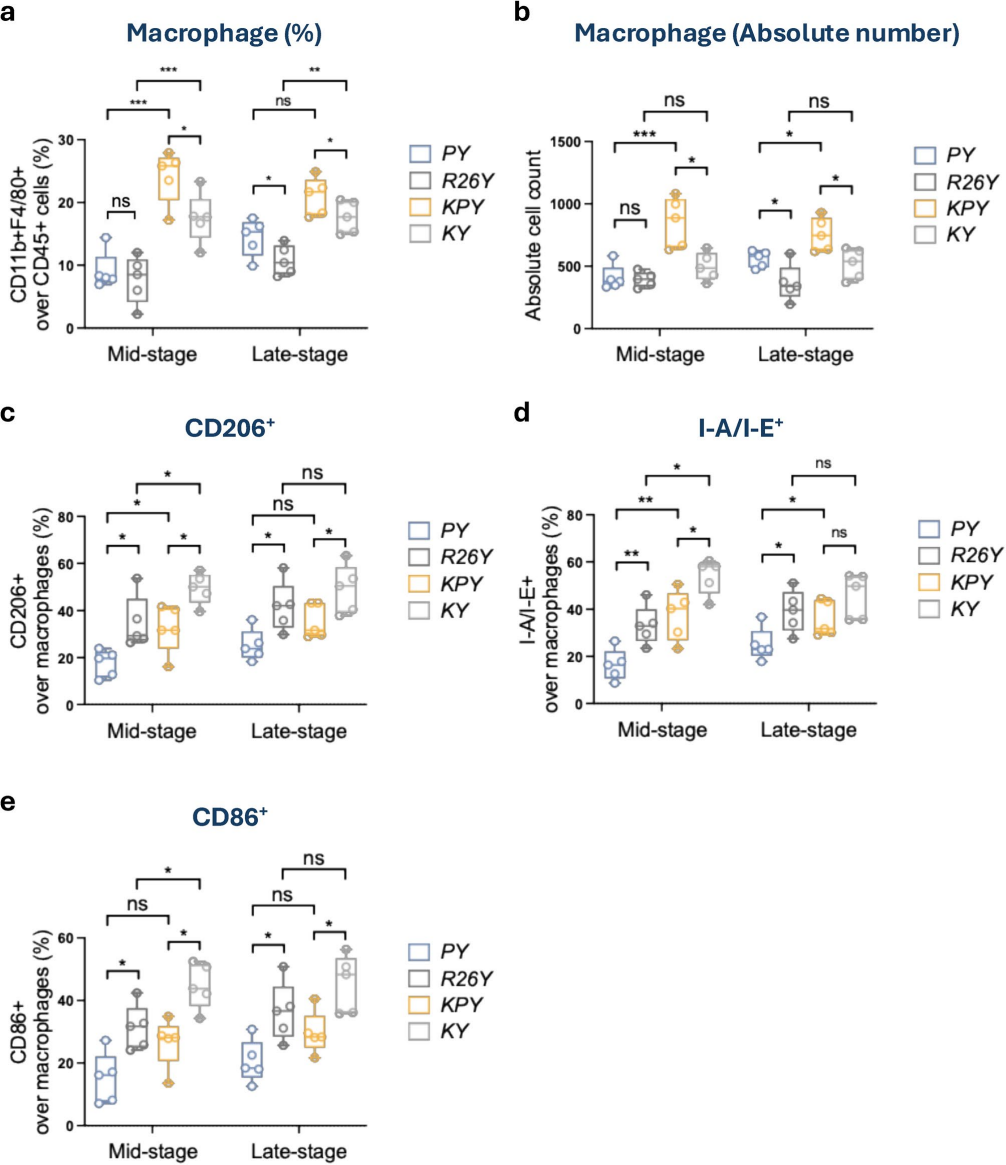
Increase in macrophage abundance driven by p53-loss was blunted in virus-induced models. (**a** and **b**) Statistical plots for FACS analysis in quantifying the percentage (a) and absolute number (b) of macrophages (CD11b^+^F4/80^+^) within the CD45^+^ compartment of mammary glands (n = 5). (**c**-**e**) Statistical plots for CD206, I-A/I-E, and CD86 expression in mammary gland macrophages (n = 5).* P* value: *p < 0.05, **p < 0.01, ***p < 0.005, ns = not significant. Data represent mean ± S.E.M.

Lastly, we examined the myeloid compartment in the mammary gland. In the TAM-induced model, induced p53-loss in luminal MECs resulted in increased macrophage infiltration (both in percentage and absolute number) at mid- and late-premalignant stages (in *KPY* vs. *KY*, Fig. 5a-b). In contrast, adenovirus-induced *PY* and *R26Y* mice exhibited a pronounced reduction in the macrophage proportion, whereas absolute macrophage numbers remained more comparable to those observed in TAM-induced mice (Fig. 5a-b). These macrophage changes appeared to be driven primarily by adenoviral infection rather than p53 status. Nevertheless, related to p53-loss, a modest late-stage increase in macrophage abundance was also detected in adenovirus-induced *PY* mice (Fig. 5a-b). Despite these quantitative differences, macrophage polarization marker expression (e.g., CD206, CD86, and I-A/I-E) remained largely similar between adenovirus-induced and TAM-induced models (Fig. 5c-e). However, we observed a trend toward reduced CD206^+^, MHC-II^+^ (i.e., I-A/I-E^+^), or CD86^+^ macrophage subpopulations in mice with induced loss of p53 (*PY* or *KPY*) compared with their respective controls (*R26Y* or *KY*) (Fig. 5c-e).

## Discussion

In this work, by characterizing and comparing mammary gland immune cells during premalignant stages in breast cancer mouse models with the same driver and tumor outcomes but different induction approaches, we observed that transient adenoviral infection not only led to an expected initial immune response, but also reshaped the mammary gland immune microenvironment long-term. The main viral infection-induced long-lasting change was the profound increase in CD8^+^ tissue-resident T cells, thereby confounding the detection of p53 loss-induced CD8^+^ T cell responses. Moreover, transient adenoviral exposure appeared to skew CD4^+^ T cells toward IFN-γ-producing anti-virus-specific states at the mid-stage. Whether such CD4^+^ T cell state affects their response to p53-deficient MECs during premalignancy is unclear. Although compared to T cells, the myeloid compartment was less affected by the adenoviral exposure, the expansion of T cell population in the adenovirus-induced model may have compressed the myeloid compartment. In the TAM-induced *KPY* (vs. *KY*) mice, induced loss of p53 in luminal MECs led to significant increases in both the percentage and absolute number of macrophages at both mid- and late-stages of premalignancy. However, such changes were less profound in adenovirus-induced *PY* (vs. *R26Y*) mice. Nevertheless, both modeling approaches revealed a similar trend of reductions in CD206^+^, MHC-II^+^ (i.e., I-A/I-E^+^), or CD86^+^ macrophage subpopulations, correlating with induced p53-loss. Overall, this study highlights the complexity of immune cell changes when using different cancer-induction approaches and offers practical insights for selecting an appropriate induction system to achieve more accurate interpretations of immune regulation during mammary tumorigenesis and beyond.

Despite adenovirus-induced changes in the mammary gland immune landscape, adenovirus-induced *PY* and TAM-induced *KPY* mice (under similar genetic background) developed mammary tumors with a similar latency^17^. This does not necessarily suggest that virus-induced changes in mammary gland immune cells have no influence on the course of tumor development. Other factors, such as the time required for p53-deficient MECs to acquire secondary driver mutations, may play a more dominant role in determining the tumor latency. Alternatively, as a tightly regulated host defense system, virus-induced upregulation of CD8^+^ tissue-resident T cells may be off-set by changes in other immune cell subsets (e.g., macrophages), making the overall immune reactions toward p53-null mutant MECs comparable to those without the virus-induced changes.

Viral infections have been found in both normal and neoplastic human breast epithelial cells. Although infections by viruses such as Epstein-Barr virus (EBV), human cytomegalovirus (HCMV), certain human papillomaviruses (HPV), and mouse mammary tumor viruses (MMTV) are more frequently found in human breast cancers^31–34^, viral infections of normal human breast epithelial cells have also been reported^33,35^. For instance, it was reported that HCMV infection could be detected in glandular epithelium in 63% of normal adult breast cases^33^. In another study, it was shown that EBV infection occurs in breast epithelial cells but not in breast cancer cells^35^. Interestingly, similar to adenovirus, HCMV is a DNA virus that does not typically integrate its DNA into the host genome and persists in the host cell episomally^36^. It is possible that viral infections of human breast epithelial cells, even only transient, could also lead to long-last changes in the immune cell landscape in the affected breast tissues, which may have influences on the risk of breast cancer development.

Adenovirus-Cre infection is a commonly used approach to induce cancer initiation in genetically engineered mice, such as those for lung cancer^37,38^, ovarian/gynecologic cancers^39,40^, sarcoma^41,42^, and bladder cancer^43,44^. It is anticipated that transient adenoviral infection in their corresponding tissues of origin may also affect their immune landscapes, potentially leading to long-lasting immune cell changes locally. Thus, caution should be taken when interpreting immune-related data from cancer mouse models when this induction approach is used.

## Methods

### Animals

All animal experiments were conducted in accordance with the approved animal protocol (2020N000122) and overseen by the Institutional Animal Care and Use Committee (IACUC) of Brigham and Women’s Hospital (BWH). The reporting in the manuscript follows the recommendations in the ARRIVE guidelines. *Trp53*^*fl/fl*^ mice (B6.129P2- *Trp53tm1*^*Brn*^/J, strain# 008462), *Rosa26-stop-YFP* (*R26Y*) mice (B6.129X1-*Gt(ROSA)26Sor*^tm1(EYFP)Cos^/J, strain# 006148), and *K8-CreER* transgenic mice (Tg(Krt8-cre/ERT2)17Blpn/J, strain# 017947) were purchased from The Jackson Laboratory (JAX). Homozygous *Trp53*^*fl/fl*^*;R26Y*^*homo*^ mice (*PY*) were obtained by breeding *Trp53*^*fl/fl*^ mice with *R26Y* mice. *K8-CreER;Trp53*^*fl/fl*^*;R26Y*^*homo*^ mice (*KPY*) were produced by further crossing *PY* mice with *K8-CreER* transgenic mice. *K8-CreER;R26Y*^*homo*^ control mice (*KY*) were generated by breeding the *K8-CreER* allele into *R26Y*^*homo*^ mice. All mouse lines have been backcrossed into the FVB/NJ background for at least six generations.

### Mouse modeling and lineage tracing

Female mice at 8 weeks of age (~ 15–20 g of body weight) were used for lineage tracing. Cre/loxP recombination in luminal mammary epithelial cells (MECs) was induced by two approaches: adenovirus-Cre infection or tamoxifen (TAM) injection. Induced mice were analyzed at 3 or 5 months later (i.e., mid-premalignant stage: ~ 3 months post-induction, ~ 20–25 g of body weight; late-premalignant stage: ~ 5 months post-induction, ~ 25–35 g of body weight).

The *Keratin 8* (*K8*) promoter-driven Cre-expressing adenoviral vector was generated in-house and deposited at the University of Iowa Viral Vector Core Facility. The *Ad-K8-Cre* adenovirus was subsequently obtained from the same facility (WC-Li-535). The concentrated adenovirus was diluted in sterile advanced DMEM/F12 culture medium supplemented with 0.1% Bromophenol blue and 0.01 M CaCl_2_. 5 µL of the diluted adenovirus prep (10^9^ pfu/mL) or the virus dilution buffer alone was injected into each abdominal mammary gland, as previously described^22^.

For tamoxifen-induced recombination, tamoxifen (T5648, Sigma-Aldrich, Chicago, USA) was dissolved in corn oil by rotating at 37 °C overnight to a final concentration of 50 mg/mL. Mice received intraperitoneal (i.p.) injections of 5 mg tamoxifen (100 µL) every other day for two doses.

### Mammary gland single cell preparation

Mice were euthanized by carbon dioxide (CO_2_) overdose in Euthanex multi-cage chamber units, which were set to introduce 100% CO_2_ at a fill rate of 30% displacement of the chamber volume per minute with CO_2_, added to the existing air in the chamber. Both abdominal (#4) mammary glands were harvested from each individual mouse and then pooled as a single sample. Tissues were minced into fine fragments and digested in digestion media (5 mL advanced DMEM/F12 culture medium containing 2% Fetal Bovine Serum (FBS), 1% HEPES, 16.5 µg Collagenase I, and 75 µg DNase I) at 37 °C for 1 h with rotation. The resulting single-cell suspension was treated with Red Blood Cell (RBC) lysis buffer for 5 min at room temperature (RT) to deplete RBCs. After washing out the lysis buffer, the remaining cells were subjected to flow cytometry analysis.

### Flow cytometry (FACS) analysis

To assess cytokine expression via intracellular staining, an aliquot of single-cell suspension from the mammary gland digestion was incubated with a cell activation cocktail in RMPI 1640 supplemented with 10% FBS and 1% Penicillin–Streptomycin for 5 h in the presence of Brefeldin A. The remaining cells were immediately subjected to antibody staining for the surface markers.

Briefly, the cells were first incubated with fixable viability dye (1:500 dilution) for 20 min at RT in the dark to label the dead cells. After centrifugation and removal of the dye solution, the cells were incubated with anti-mouse CD16/32 antibody (1:200; Cat# 156603, clone S17011E; BioLegend, San Diego, USA) at 4 °C for 5 min to block non-specific binding. Subsequently, cells were stained with a master mix of fluorophore-conjugated antibodies against surface markers (see Table 1 for details) at 4 °C for 30 min, with dilutions as specified by the manufacturers’ protocols. After washing out the antibody dilution, the cells were resuspended and immediately analyzed by flow cytometry.

**Table 1:** FACS antibodies.

REAGENT or RESOURCE	SOURCE	CLONE#	IDENTIFIER
Rat anti-mouse CD45 (BV605)	Biolegend	3—F11	Cat# 103155
Rat anti-mouse TER-119 (BV605)	Biolegend	TER-119	Cat# 116239
Rat anti-mouse CD31 (BV605)	Biolegend	390	Cat# 102427
Rat anti-mouse CD24 (PE)	Biolegend	M1/69	Cat# 101808
Hamster anti-mouse CD29 (APC)	Biolegend	HMβ1-1	Cat# 102216
Rat anti-mouse CD8a (BUV395)	BD	53–6.7	Cat# 565968
Hamster anti-mouse CD3 (BUV737)	BD	500A2	Cat# 741716
Rat anti-mouse CD4 (BV711)	Biolegend	RM4-5	Cat# 100549
Rat anti-mouse NKp46 (BV650)	Biolegend	29A1.4	Cat# 137635
Hamster anti-mouse CD69 (PE/Cyanine7)	Biolegend	H1.2F3	Cat# 104512
Rat anti-mouse PD-1 (PE/Cyanine5)	Biolegend	29F.1A12	Cat# 135255
Rat anti-mouse CD45 (Alexa Fluor 700)	Biolegend	30-F11	Cat# 103128
Rat anti-mouse IFN-γ (BV605)	Biolegend	XMG1.2	Cat# 505839
Rat anti-mouse CD73 (BV605)	Biolegend	TY/11.8	Cat# 127215
Mouse anti-mouse CD103 (BV421)	Biolegend	QA17A24	Cat# 156915
Rat anti-mouse F4/80 (BUV395)	BD	T45-2342	Cat# 565614
Rat anti-mouse CD19 (BUV737)	BD	1D3	Cat# 612782
Rat anti-mouse CD11b (BV650)	Biolegend	M1/70	Cat# 101239
Rat anti-mouse I-A/I-E (BV605)	Biolegend	M5/114.15.2	Cat# 107639
Hamster anti-mouse CD11c (BV510)	Biolegend	N418	Cat# 117337
Rat anti-mouse Ly-6G (BV421)	Biolegend	1A8	Cat# 127627
Rat anti-mouse CD86 (PerCP/Cyanine 5.5)	Biolegend	GL-1	Cat# 105027
Rat anti-mouse CD206 (PE/Dazzle 594)	Biolegend	C068C2	Cat# 141732
Zombie NIR Fixable Viability Kit	Biolegend	N/A	Cat# 423106
Zombie UV Fixable Viability Kit	Biolegend	N/A	Cat# 423108

For intracellular staining, pre-activated cells were harvested and processed for surface marker staining first, as described above. Then, the cells were fixed and permeabilized using the True-Nuclear Transcription Factor Buffer Set (Cat# 424401, BioLegend), and subsequently labeled with the corresponding FACS antibodies.

FACS data were acquired on a BD LSR II or BD Symphony A5 analyzer (BD Biosciences, San Jose, USA) and analyzed using Flowjo software (10.10.0). Gating strategies are provided in Supplementary Fig. 3.

### Statistics

The results were presented as mean ± S.E.M. Student’s *t* tests were used for the comparisons between two groups. Comparisons of multiple groups were performed by two-way ANOVA, and p-values were adjusted using Tukey’s method. No randomization or blinding was used in the in vivo studies. Differences are considered to be significant for *p < 0.05, **p < 0.01, and ***p < 0.005.

## Data Availability

All data generated and analyzed in this study are included in this published article and its Supplementary files.
